# Impact of a Combined Continuous Glucose Monitoring–Digital Health Solution on Glucose Metrics and Self-Management Behavior for Adults With Type 2 Diabetes: Real-World, Observational Study

**DOI:** 10.2196/47638

**Published:** 2023-09-11

**Authors:** Abhimanyu B Kumbara, Anand K Iyer, Courtney R Green, Lauren H Jepson, Keri Leone, Jennifer E Layne, Mansur Shomali

**Affiliations:** 1 Welldoc, Inc Columbia, MD United States; 2 Dexcom, Inc San Diego, CA United States

**Keywords:** type 2 diabetes, digital health, continuous glucose monitoring, artificial intelligence, glycemic outcomes, engagement, digital health intervention, mHealth, diabetes management

## Abstract

**Background:**

The BlueStar (Welldoc) digital health solution for people with diabetes incorporates data from multiple devices and generates coaching messages using artificial intelligence. The BlueStar app syncs glucose data from the G6 (Dexcom) real-time continuous glucose monitoring (RT-CGM) system, which provides a glucose measurement every 5 minutes.

**Objective:**

The objective of this real-world study of people with type 2 diabetes (T2D) using the digital health solution and RT-CGM was to evaluate change in glycemic control and engagement with the program over 3 months.

**Methods:**

Participants were current or former enrollees in an employer-sponsored health plan, were aged 18 years or older, had a T2D diagnosis, and were not using prandial insulin. Outcomes included CGM-based glycemic metrics and engagement with the BlueStar app, including logging medications taken, exercise, food details, blood pressure, weight, and hours of sleep.

**Results:**

Participants in the program that met our analysis criteria (n=52) were aged a mean of 53 (SD 9) years; 37% (19/52) were female and approximately 50% (25/52) were taking diabetes medications. The RT-CGM system was worn 90% (SD 8%) of the time over 3 months. Among individuals with suboptimal glycemic control at baseline, defined as mean glucose >180 mg/dL, clinically meaningful improvements in glycemic control were observed, including reductions in a glucose management indicator (–0.8 percentage points), time above range 181-250 mg/dL (–4.4 percentage points) and time above range >250 mg/dL (–14 percentage points; all *P*<.05). Time in range 70-180 mg/dL also increased by 15 percentage points (*P*=.016) in this population, which corresponds to an increase of approximately 3.5 hours per day in the target range. Over the 3-month study, 29% (15/52) of participants completed at least one engagement activity per week. Medication logging was completed most often by participants (23/52, 44%) at a rate of 12.1 (SD 0.8) events/week, and this was closely followed by exercise and food logging.

**Conclusions:**

The combination of an artificial intelligence–powered digital health solution and RT-CGM helped people with T2D improve their glycemic outcomes and diabetes self-management behaviors.

## Introduction

Type 2 diabetes (T2D) affects over 10% of the US population [1], and management of it is complex and challenging. As a result, only about half of individuals diagnosed with diabetes are meeting the American Diabetes Association (ADA) treatment target of glycated hemoglobin (HbA_1C_) <7% [1]. Several modifiable lifestyle factors that contribute to suboptimal glycemia include the challenge for a person with diabetes to easily and diligently execute all self-management behaviors as outlined in the Association of Diabetes Care and Education Specialists framework (ADCES-7) [2], including the management of medications, glucose, activity, diet, coping, risk, and problem-solving. The prevalence of infrequent and intermittent fingerstick blood glucose testing does not easily allow for a “teachable moment” whereby an individual with T2D can correlate cause (ie, a specific behavior) and effect (ie, glucose level).

In recent years, digital health solutions that aim to improve the lives of people with T2D have grown in both number and scale [3]. These digital health solutions include a smartphone app that aids in diabetes decision support and provides insights. Some also provide items and features such as connected blood glucose meters (BGMs), continuous glucose monitors (CGMs), medication management support, live coaching, artificial intelligence (AI) or other data-driven insights, or logs where users can track events such as physical activity, sleep, and medication use.

The ADA Standards of Medical Care in Diabetes recommends the use of digital health technology in treating diabetes for some individuals [4]. Systematic reviews found that the majority of technology-enabled diabetes management interventions are associated with HbA_1C_ reductions [5,6] and can improve self-efficacy, leading to greater self-confidence [5]. This was also seen in studies with multiple intervention types [7] that used connected BGMs [8] and in a digital diabetes management program that offers personalized educational content [9].

BlueStar (Welldoc) is a US Food and Drug Administration (FDA)–cleared digital health solution that guides individuals through the journey of living with diabetes by enabling them to self-manage their care while enhancing connections to their health care team. BlueStar is indicated for use by health care providers and their patients—aged 18 years and older—who have type 1 diabetes or T2D. The BlueStar app’s novelty manifests in many dimensions. First, it supports all ADCES-7 self-management behaviors through scalable, evidence-based, and FDA-cleared software as a medical device. Second, it provides AI-driven motivational, behavioral, or educational coaching, both in real time and longitudinally. The AI insights identify key patterns and areas of concern that can be escalated to a health care provider, which may help overcome clinical inertia.

The use of real-time continuous glucose monitoring (RT-CGM) has the potential to overcome the challenge associated with infrequent and intermittent blood glucose testing. RT-CGM systems provide a glucose measurement of the interstitial fluid every 1-5 minutes and contain programmable alerts and alarms that warn users of current or impending glycemic excursions. The combination of RT-CGM and digital health and its combined effect on self-management behaviors and outcomes warrants further study. The aims of this real-world study of people with T2D using a digital health solution (BlueStar) and RT-CGM (G6; Dexcom) were to assess the changes in glycemic control and in engagement with the digital health solution over 3 months.

## Methods

### Study Details and Participants

This single-arm, retrospective, real-world study evaluated the change in glycemic control and engagement in people with T2D that participated in the combined BlueStar digital health and Dexcom RT-CGM program for 3 months. As this was a feasibility study under real-world conditions, the sample size was not predetermined. Participants were recruited from 3 health care clinics in the United States and were current or former enrollees in an employer-sponsored health program. Study enrollment occurred between February 2021 and January 2022.

Eligibility criteria included being aged 18 years or older, being willing to use the BlueStar app and the Dexcom G6 RT-CGM system, and having a diagnosis of T2D treated with basal insulin, oral medications, noninsulin injectables, or lifestyle management. Key exclusion criteria included prandial insulin use, pregnancy, cancer, dialysis, or the presence of a major psychiatric disorder.

Initial enrollment included downloading the BlueStar and Dexcom G6 mobile apps (compatible with Apple and Android phones), provision of a CGM transmitter and three 10-day sensors (1 month’s supply), and optional training on CGM insertion and use. Participants were required to return to the clinic each month to obtain additional sensors. The BlueStar app is an integrated digital health platform that can sync with numerous devices, provide personalized feedback and digital coaching to its users, and help users track their medication, sleep, exercise, and other health behaviors. The Dexcom G6 mobile app allows users to view their glucose levels in real time with updates every 5 minutes. Trend arrows and access to retrospective data using Clarity reporting software (Dexcom, Inc) can help users identify short- and long-term patterns in their glucose levels.

### Ethics Approval

Ethical review and full waiver of Health Insurance Portability and Accountability Act (HIPAA) authorization were obtained from Advarra Institutional Review Board (Pro00069142).

### Outcome Measures

#### Glucose Metrics

The primary outcome was the change in glycemic control after 3 months, which was assessed from CGM data that participants uploaded to the Dexcom app. The International Consensus on Time in Range (TIR) guidelines were used to calculate standardized CGM metrics, including change in mean glucose, glucose management indicator (GMI), coefficient of variation (CV), percentage of TIR (70-180 mg/dL), percentage of time above range (TAR) (level 1: 181-250 mg/dL or level 2: >250 mg/dL), and percentage of time below range (TBR; <70 mg/dL) [10]. The proportion of days with CGM use was also assessed.

#### Engagement

Engagement with BlueStar and its lifestyle tracking features was also analyzed. All participant interaction with the BlueStar app was recorded by the app software, including opening the app, logging activities, and accessing educational materials. The engagement outcome quantified “meaningful” activities: logging medication-taking or entering food details, blood pressure, weight, hours of sleep, or exercise. Instances of simply opening the app were excluded. Overall engagement was numerically defined as the proportion of participants with ≥1 engagement activity per week in the 3-month window.

### Statistical Analysis

The program originally enrolled 122 participants. To be included in the analysis cohort, participants were required to have 10 contiguous calendar days of CGM readings with 70% data sufficiency within 30 days from activation on the BlueStar platform and a follow-up measurement of 10 contiguous calendar days of CGM readings with 70% data sufficiency between 80 and 110 days after activation on the BlueStar platform. After excluding participants treated with bolus insulin and applying the baseline and follow-up CGM data sufficiency criteria, the final analysis cohort was 52 participants. Average engagement per user per week was calculated for users who logged medication-taking, exercise, food, weight, sleep, and blood pressure events. To be included in the engagement analysis, a user had to have ≥1 engagement for a given event between the baseline and follow-up glycemic outcome measurement periods as noted above.

CGM metrics and engagement measures were then calculated for two population segments: (1) participants with mean baseline glucose >180 mg/dL (suboptimal control) and (2) participants with mean baseline glucose ≤180 mg/dL. The cutoff of 180 mg/dL was chosen because it is the upper bound of the International Consensus TIR guideline metric [10]. Given the small sample size, we tested the CGM data for normality using the Shapiro-Wilk test. The data were not normally distributed so a Wilcoxon signed rank test was used to evaluate 2 distributions of baseline and follow-up groups. The between-groups difference in the rates of engagement with BlueStar app features was evaluated using the Mann-Whitney *U* test. To test for significant differences in demographic information, we performed a 2-proportion *z* test for gender and medication regimen and a 2-tailed Welch *t* test for age, baseline GMI, and baseline mean glucose. Significance for all statistical tests was defined as *P*<.05.

## Results

### Overview

**Figure 1:**
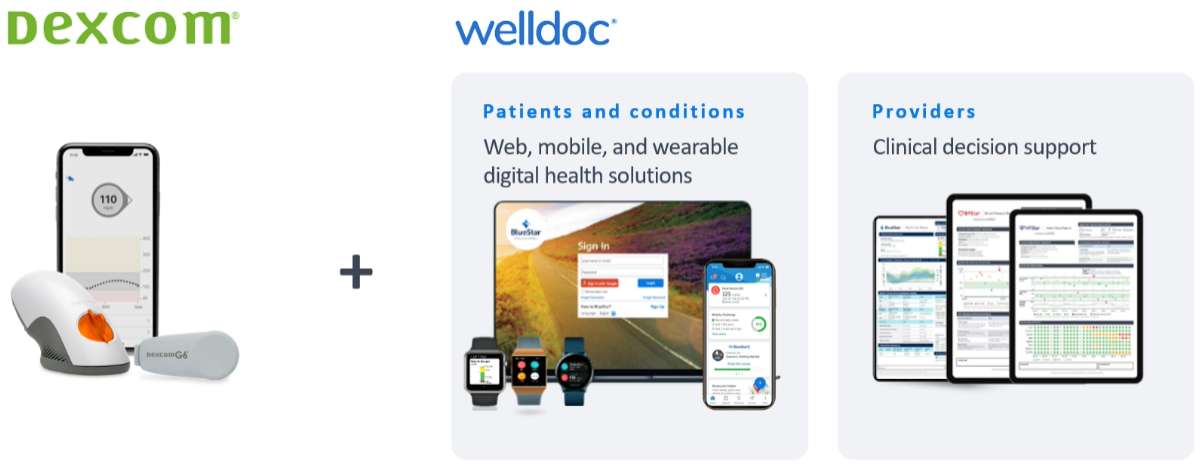
Dexcom G6 real-time continuous glucose monitoring system and Welldoc BlueStar digital health solution.

A total of 122 participants enrolled in the study; 94 initiated RT-CGM and 52 met the data sufficiency requirements at 3 months. There were no significant differences in age or gender between those who met the data sufficiency requirements (n=52) and those who did not (n=42). The combined digital health solution/RT-CGM program is depicted in Figure 1. Baseline demographic and clinical characteristics of participants are presented in Table 1. Participants (n=52) were 37% (19/52) female with an average age of 53 (SD 9) years. In their initial sensor session, 65% (34/52) of participants had a mean glucose ≤180 mg/dL and 35% (18/52) had a mean glucose >180 mg/dL.

**Table 1:** Participant characteristics.

Baseline characteristics			Overall (n=52)		Baseline glucose ≤180 mg/dL (n=34)		Baseline glucose >180 mg/dL (n=18)		*P* value^a^
Age (years), mean (SD)			53 (9)		53 (7)		50 (11)		.07
Female, n (%)			19 (37)		14 (41)		5 (28)		.35
GMI^b^, mean (SD)			7.5 (1.1)		6.8 (0.5)		8.7 (0.9)		<.001
Mean glucose (mg/dL), mean (SD)			174 (47)		147 (20)		227 (36)		<.001
**Diabetes medication regimen, n (%)**									
	Basal insulin	6 (12)		3 (9)		4 (22)		.18	
	GLP-1 RA^c^	3 (6)		2 (6)		1 (6)		.96	
	Oral medications	16 (31)		13 (38)		3 (17)		.11	
	No medications	26 (50)		16 (47)		10 (56)		.56	

^a^*P* values describe the comparisons between the ≤180 mg/dL cohort and the >180 mg/dL cohort. Between-group differences were tested using the 2-proportion *z* test for gender and medication regimen and a 2-tailed Welch *t* test for age, glucose management indicator (GMI), and mean glucose.

^b^GMI: glucose management indicator.

^c^GLP-1 RA: glucagon-like peptide 1 receptor agonist.

### Glycemic Outcomes

The mean proportion of days with sensor wear was 90% (SD 8%). Glycemic outcomes for the 3-month study are presented in Table 2. Among individuals with baseline mean glucose >180 mg/dL, significant improvements in glycemic metrics were observed. Specifically, GMI decreased by 0.8 (IQR –1.0 to 0.1) percentage points, TIR increased by 15 (IQR 1-47) percentage points, and TAR 181-250 mg/dL and >250 mg/dL decreased by 4.4 (IQR –20.2 to 2.1) and 14 (IQR –18.3 to 1.4) percentage points, respectively (all *P*<.05). This TIR improvement corresponds to an increase of over 3.5 hours per day in target range and is clinically meaningful [10]. Glycemic control among participants with a baseline mean glucose ≤180 mg/dL was maintained [10]. The proportion meeting the international consensus guidelines of >70% TIR [10] increased from 0% to 28% among individuals with baseline glucose >180 mg/dL.

**Table 2:** Change in continuous glucose monitoring metrics from baseline at 3-month follow-up stratified by baseline mean glucose.

Continuous glucose monitoring metric			Overall					Baseline glucose ≤180 mg/dL							Baseline glucose >180 mg/dL						
			Baseline	Follow-up		Change		Baseline		Follow-up		Change		Baseline			Follow-up		Change		
**Glycemic control, median (IQR)**																					
	Mean glucose (mg/dL)	164 (138 to 198)		155 (130 to 188)	–9 (–35 to 17)		145 (126 to 164)		141 (127 to 181)		–4 (–11 to 22)		221 (198 to 233)			187 (160 to 214)		–34 (–42 to 6)^a^			
	GMI^b^ (%)	7.2 (6.6 to 8)		7 (6.4 to 7.8)	–0.2 (–0.8 to 0.4)		6.8 (6.3 to 7.2)		6.7 (6.3 to 7.6)		–0.1 (–0.3 to 0.5)		8.6 (8 to 8.9)			7.8 (7.1 to 8.4)		–0.8 (–1 to 0.1)^a^			
	CV^c^ (%)	22 (19.7 to 24.7)		22.2 (19.4 to 25.9)	0.2 (–2.2 to 2.9)		22 (19.7 to 24.7)		21 (18.8 to 25.5)		–1 (–2.1 to 2.1)		21.8 (20 to 25.6)			22.9 (20.2 to 27.7)		1.1 (–2.4 to 3.7)			
**Time in, above, or below range (%), median (IQR)**																					
	TIR^d^ 70-180 mg/dL	71.1 (31 to 88)		72.2 (47 to 92)	1.1 (–14 to 19)		82 (72 to 94)		83 (56 to 94)		1 (–19 to 7)		21 (15 to 38)			36 (25 to 72)		15 (1 to 47)^a^			
	TAR^e^ 181-250 mg/dL	23.3 (10 to 40)		21 (6 to 38)	–2.3 (–10 to 6)		16.2 (5.1 to 24.3)		13.1 (6.4 to 36.3)		–3 (–7.2 to 11.1)		40.2 (33.1 to 56.3)			36.3 (23.1 to 48.2)		–4.4 (–20.2 to 2.1)^a^			
	TAR >250 mg/dL	2.1 (0.3 to 12.1)		2.3 (0.1 to 11.3)	–0.2 (–5.2 to 4.4)		1.2 (0.1 to 2.2)		0.2 (0.3 to 5.2)		1 (0.1 to 5.3)^a^		25 (11.1 to 43.4)			11 (1.2 to 27.2)		–14 (–18.3 to 1.4)^a^			
	TBR^f^ <70 mg/dL	0.2 (0.1 to 0.6)		0.4 (0.2 to 1.3)	0.2 (–0.2 to 1.1)		0.2 (0.1 to 0.8)		0.6 (0.2 to 0.7)		0.4 (–0.1 to 0.9)		0.2 (0.1 to 0.5)			0.5 (0.2 to 1.5)		0.3 (0.1 to 0.9)			

^a^*P*<.05; significance of the within-group differences was tested using the Wilcoxon signed rank test.

^b^GMI: glucose management indicator.

^c^CV: coefficient of variation.

^d^TIR: time in range.

^e^TAR: time above range.

^f^TBR: time below range.

### Engagement

The proportion of individuals with at least one engagement activity per week over the 3-month study period was 29% (15/52). Logging of specific engagement activities overall and stratified by baseline mean glucose is shown in Table 3. The weekly engagement rate per user who logged events is shown in Table 4. Among individuals who logged events, there was a significantly higher total rate of event logging in the cohort with baseline mean glucose ≤180 mg/dL (*P*=.006). Medication-taking was logged most often in both cohorts, closely followed by exercise and food logging. Between the 2 cohorts, there were significantly higher rates of medication (*P*<.001), exercise (*P*<.001), food (*P*=.007), and sleep (*P*<.001) logging among participants with baseline mean glucose ≤180 mg/dL.

**Table 3:** Summary of event logging.

Participant engagement	Overall (n=52), n (%)	Baseline mean glucose ≤180 mg/dL (n=34), n (%)	Baseline mean glucose >180 mg/dL (n=18), n (%)	*P* value^a^
Medications	23 (44)	15 (44)	8 (44)	>.99
Exercise	21 (40)	13 (38)	8 (44)	.70
Food	19 (37)	12 (35)	7 (39)	.80
Weight	18 (35)	12 (35)	6 (33)	.90
Sleep	16 (31)	11 (32)	5 (28)	.80
Blood pressure	7 (13)	6 (18)	1 (6)	.20

^a^*P* values were calculated using a 2-proportion *z* test and describe comparisons between the ≤180 mg/dL cohort and the >180 mg/dL cohort.

**Table 4:** Average engagement rate among participants who logged events.

Average engagement (per user per week)	Overall, mean (SD)	Baseline mean glucose ≤180 mg/dL, mean (SD)	Baseline mean glucose >180 mg/dL, mean (SD)	*P* value^a^
Medications	12.1 (0.8)	15.2 (1.5)	6.4 (0.7)	<.001
Exercise	5.0 (0.2)	5.5 (0.4)	4.4 (0.4)	<.001
Food	3.8 (0.6)	4.5 (1.1)	2.4 (0.7)	.007
Sleep	2.4 (0.2)	2.5 (0.2)	2.1 (0.4)	<.001
Blood pressure	1.1 (0.2)	1.2 (0.3)	0.2 (N/A^b^)	N/A
Weight	0.7 (0.1)	0.7 (0.1)	0.6 (0.1)	.62
Total	25.0	29.7	16.1	.006

^a^*P* values were calculated using the Mann-Whitney U test and describe the comparisons between the ≤180 mg/dL cohort and the >180 mg/dL cohort.

^b^N/A: not applicable (not enough data for statistical analysis).

## Discussion

### Principal Results

In this real-world study, participation in a digital health program combined with RT-CGM use was associated with significant improvements in glycemic control in adults with T2D and suboptimal glycemic control at baseline. This included a significant decrease in mean glucose and a clinically meaningful increase in TIR of approximately 15 percentage points, corresponding to an increase of approximately 3.5 hours per day time in the target glucose range [10]. Glycemic control was maintained in the group of participants meeting glycemic targets (mean glucose ≤180 mg/dL) at baseline. There was high RT-CGM use and regular engagement with the BlueStar app. Regardless of baseline glucose control, a similar proportion of participants engaged with the BlueStar app and logged self-management events.

### Comparison With Prior Work

There are a variety of digital health programs that incorporate CGM. Some use only live lifestyle coaching; others use live coaching and telemedicine visits; some exclusively use AI-based lifestyle programs; and others use hybrid models of AI-based and live coaching, with or without telemedicine visits. Studies of these programs have reported glycemic and other outcomes; however, it is not possible to directly compare the results to those in this study due to differences in the program models for delivering diabetes educational support and coaching, live clinician management, and CGM wear patterns (continuous vs intermittent wear) [8,9,11-17]. For example, a study by Majithia et al [12] of a digital health program incorporating telemedicine visits found a 10.2 percentage point increase in TIR at 4-month follow-up among participants with suboptimally controlled T2D versus a 15 percentage point increase in this study. However, CGM was worn nearly continuously in this study versus intermittent use in the report by Majithia et al [12]. A retrospective study of people with T2D using CGM continuously as part of an intensive diabetes management program also reported a significant decrease in CGM-derived mean glucose (147.4, SD 59.1 mg/dL to 122.6, SD 33.3 mg/dL; *P*<.001), and significant improvement in HbA_1C_, insulin resistance, and fasting blood glucose at 90 days [15]. As in our study, other studies of combined telehealth and CGM programs in people with T2D have reported greater glycemic improvements among those with a higher baseline value and maintenance of glycemic control in those meeting targets at baseline [13,14].

Beyond glycemic outcomes, participation in digital health programs incorporating CGM has been associated with other beneficial outcomes. Participants in telehealth programs that incorporated RT-CGM reported an increased understanding of their diabetes [13], lower diabetes distress [16,18], and improved quality of life [16]. In a previous study of users in a combined BlueStar and connected BGM application, emergency department visits decreased by 30% and costs decreased by 55% [11].

Engagement metrics are not commonly reported in studies on digital health or telehealth solutions. In one study, two-thirds of participants in a telehealth program incorporating RT-CGM had at least one coaching interaction, with most having greater than 4 during the 26-week follow-up period [14]. In another report, among users of a telehealth mobile app, 84% used coaching and 13% used telemedicine at least once [17]. Notably, those who used CGM had some of the largest improvements at follow-up [17].

It is noteworthy that while the proportion of participants that logged events was similar between groups, among those who logged events, the weekly rate was higher in the group with better glycemic control at baseline. This is reported in other studies where individuals with good glycemic control maintain it through the intervention [13,15]. While our study did not query participants on their engagement behaviors, we hypothesize that individuals with higher average baseline glucose primarily focused on their glucose data and related insights, whereas individuals with better glycemic control had increased availability to log other behaviors. This will be a topic of future research.

The BlueStar digital health solution is designed for high scalability in its use of AI-powered insights rather than live, lifestyle coaching with diabetes educators or telemedicine visits with clinicians. These insights from RT-CGM data can aid users in successful problem-solving, a critical ADCES7 self-care behavior [2], and potentially lead to behavioral change in other ADCES7 self-care behaviors. For example, glucose changes following exercise, a healthy meal, or taking medication as prescribed could clarify the importance of these diabetes self-management behaviors. The combination of a digital health solution and RT-CGM allows for personalized problem-solving and is likely to lead to improvements in diabetes management.

### Strengths and Limitations

Strengths of this study include the significant improvements in participant outcomes, the use of a real-world, outpatient setting, and the sustainability of the intervention related to the scalability of the digital health solution. Limitations include the all-US population covered under one employee health plan, limited demographic information (such as education level), and the modest sample size, which could have limited statistical significance. In addition, the effect of the digital health solution or RT-CGM system individually could not be quantified in this single-arm study design. Longer studies are needed to evaluate the durability of outcomes. We also acknowledge the potential attrition bias with the use of a 70% data sufficiency requirement; however, our aim was to assess the use of the combined digital health and RT-CGM program and determine its efficacy.

### Conclusions

This study suggests that engagement with a combination of RT-CGM and an AI-driven digital health solution helps users improve their glycemic metrics. Those with the highest baseline glycemic metrics were more likely to see significant, meaningful improvements. Behavioral change stemming from AI-derived insights and interaction with RT-CGM data likely contributes to these improvements without the need for live coaching or telehealth visits.

## Abbreviations

AI: artificial intelligence
ADA: American Diabetes Association
ADCES: Association of Diabetes Care and Education Specialists
AI: artificial intelligence
BGM: blood glucose meter
CGM: continuous glucose monitor
CV: coefficient of variation
GMI: glucose management indicator
HbA_1C_: glycated hemoglobin FDA: Food and Drug Administration
HIPAA: Health Insurance Portability and Accountability Act
RT-CGM: real-time continuous glucose monitor
T2D: type 2 diabetes
TAR: time above range
TBR: time below range
TIR: time in range

## References

[1] National diabetes statistics report. Centers for Disease Control and Prevention.

[2] Kolb L, Association of Diabetes Care and Education Specialists (2021). An effective model of diabetes care and education: the ADCES7 self-care behaviors™. Sci Diabetes Self Manag Care.

[3] Levine BJ, Close KL, Gabbay RA (2020). Reviewing U.S. connected diabetes care: the newest member of the team. Diabetes Technol Ther.

[4] ElSayed NA, Aleppo G, Aroda VR, Bannuru RR, Brown FM, Bruemmer D, Collins BS, Hilliard ME, Isaacs D, Johnson EL, Kahan S, Khunti K, Leon J, Lyons SK, Perry ML, Prahalad P, Pratley RE, Seley JJ, Stanton RC, Gabbay RA, on behalf of the American Diabetes Association (2023). 7. Diabetes technology: standards of care in diabetes-2023. Diabetes Care.

[5] Bonoto BC, de Araújo VE, Godói IP, de Lemos LLP, Godman B, Bennie M, Diniz LM, Junior AAG (2017). Efficacy of mobile apps to support the care of patients with diabetes mellitus: a systematic review and meta-analysis of randomized controlled trials. JMIR Mhealth Uhealth.

[6] Greenwood DA, Gee PM, Fatkin KJ, Peeples M (2017). A systematic review of reviews evaluating technology-enabled diabetes self-management education and support. J Diabetes Sci Technol.

[7] Quinn CC, Shardell MD, Terrin ML, Barr EA, Ballew SH, Gruber-Baldini AL (2011). Cluster-randomized trial of a mobile phone personalized behavioral intervention for blood glucose control. Diabetes Care.

[8] Mora P, Buskirk A, Lyden M, Parkin CG, Borsa L, Petersen B (2017). Use of a novel, remotely connected diabetes management system is associated with increased treatment satisfaction, reduced diabetes distress, and improved glycemic control in individuals with insulin-treated diabetes: first results from the personal diabetes management study. Diabetes Technol Ther.

[9] Zimmermann G, Venkatesan A, Rawlings K, Scahill MD (2021). Improved glycemic control with a digital health intervention in adults with type 2 diabetes: retrospective study. JMIR Diabetes.

[10] Battelino T, Danne T, Bergenstal RM, Amiel SA, Beck R, Biester T, Bosi E, Buckingham BA, Cefalu WT, Close KL, Cobelli C, Dassau E, DeVries JH, Donaghue KC, Dovc K, Doyle FJ, Garg S, Grunberger G, Heller S, Heinemann L, Hirsch IB, Hovorka R, Jia W, Kordonouri O, Kovatchev B, Kowalski A, Laffel L, Levine B, Mayorov A, Mathieu C, Murphy HR, Nimri R, Nørgaard K, Parkin CG, Renard E, Rodbard D, Saboo B, Schatz D, Stoner K, Urakami T, Weinzimer SA, Phillip M (2019). Clinical targets for continuous glucose monitoring data interpretation: recommendations from the International Consensus on Time in Range. Diabetes Care.

[11] Shearer D, Iyer A, Peeples M (2021). A payer digital health study shows scalable approach to cost savings and outcomes. J Diabetes Sci Technol.

[12] Majithia AR, Kusiak CM, Lee AA, Colangelo FR, Romanelli RJ, Robertson S, Miller DP, Erani DM, Layne JE, Dixon RF, Zisser H (2020). Glycemic outcomes in adults with type 2 diabetes participating in a continuous glucose monitor-driven virtual diabetes clinic: prospective trial. J Med Internet Res.

[13] Bergenstal RM, Layne JE, Zisser H, Gabbay RA, Barleen NA, Lee AA, Majithia AR, Parkin CG, Dixon RF (2021). Remote application and use of real-time continuous glucose monitoring by adults with type 2 diabetes in a virtual diabetes clinic. Diabetes Technol Ther.

[14] Kamrudin S, Clark C, Mondejar JP, Vargas MF, Thompson N, Macfarlane E, Defail A, Oechler AL, Cook DJ (2022). 695-P: engagement and glycemic outcomes over 24 weeks among new level2 members. Diabetes.

[15] Shamanna P, Saboo B, Damodharan S, Mohammed J, Mohamed M, Poon T, Kleinman N, Thajudeen M (2020). Reducing HbA1c in type 2 diabetes using digital twin technology-enabled precision nutrition: a retrospective analysis. Diabetes Ther.

[16] Gal RL, Cohen NJ, Kruger D, Beck RW, Bergenstal RM, Calhoun P, Cushman T, Haban A, Hood K, Johnson ML, McArthur T, Olson BA, Weinstock RS, Oser SM, Oser TK, Bugielski B, Strayer H, Aleppo G (2020). Diabetes telehealth solutions: improving self-management through remote initiation of continuous glucose monitoring. J Endocr Soc.

[17] Chen Z, Birse CE, Fragala M, Mcphaul MJ, Bare L (2022). SU3024 / 3024—independent evaluation of effects of a virtual employer-sponsored type 2 diabetes management program on blood glucose.

[18] Polonsky WH, Layne JE, Parkin CG, Kusiak CM, Barleen NA, Miller DP, Zisser H, Dixon RF (2020). Impact of participation in a virtual diabetes clinic on diabetes-related distress in individuals with type 2 diabetes. Clin Diabetes.

